# Informing the Future: Cancer Prevention and Diagnosis Beliefs Among Older Latino Immigrants

**DOI:** 10.1093/geroni/igaa057.1057

**Published:** 2020-12-16

**Authors:** Iraida Carrion, Malinee Neelamegam, Jane Roberts

**Affiliations:** 1 University of South Florida, Tampa, Florida, United States; 2 Yale School of Public Health, New Haven, Connecticut, United States; 3 University of South Florida, Sarasota, Florida, United States

## Abstract

Despite emerging research on Latinos and cancer (Carrion et al., 2018), there are no data regarding beliefs about cancer prevention and diagnosis in older Latinos residing in Central Florida. Similarly, to the US in general, Central Florida’s older Latino population is growing. Compared with other national samples, Latino immigrants in the southern U.S. report poorer health than in other regions (Siegel et al., 2015). Using convenience sampling (N = 168), univariate analysis was done to recognize the study population’s characteristics. Frequencies were assessed to understand participants’ responses to questions on cancer-related attitudes. The effects of age, country of origin, length of stay in the U.S., and marital status were assessed using logistic regression. Of the 168 individuals in the study, 34.5% were male with a mean age of 67.9, and a majority had at least a high school education and 25.8 years residing in the U.S. The participants were aware that tobacco use can cause cancer (93.5%) and that smoking affects the smoker as well as their family members (84.5%). They were also aware that mammograms facilitate early diagnosis of breast cancer (81.5%) and of the association between prolonged sun exposure and skin cancer (86.9%). However, specific knowledge about early diagnosis was low. Only 29.2% of participants knew that breast cancer can be diagnosed early, which was similar to the response toward early diagnosis of prostate cancer (24.4%). Among the participants, 26.2% were categorized as having poor knowledge of cancer prevention.

